# Supplementary material for: Quantifying the differential functional behavior between the medial and lateral meniscus after posterior meniscus root tears
Source: PLoS One. 2021 Nov 10;16(11):e0259678. doi: 10.1371/journal.pone.0259678 (PMC8580232; doi:10.1371/journal.pone.0259678)
Supplement: S1 Table — yr = age of the specimen in years. (DOCX) [file pone.0259678.s001.docx]

**S1 Table. Descriptive data of tested specimens.**

| **Specimen** | **Age (yr)** | **Sex** | **Side** | **Meniscus** |
| --- | --- | --- | --- | --- |
| **1** | 81 | Female | Left | Medial |
| **2** | 66 | Female | Right | Medial |
| **3** | 71 | Female | Right | Medial |
| **4** | 71 | Male | Right | Medial |
| **5** | 81 | Female | Right | Medial |
| **6** | 62 | Male | Right | Medial |
| **7** | 84 | Female | Right | Lateral |
| **8** | 84 | Male | Left | Lateral |
| **9** | 62 | Male | Left | Lateral |
| **10** | 69 | Male | Left | Lateral |
| **11** | 36 | Male | Right | Lateral |
