# Supplementary material for: Quantifying the differential functional behavior between the medial and lateral meniscus after posterior meniscus root tears
Source: PLoS One. 2021 Nov 10;16(11):e0259678. doi: 10.1371/journal.pone.0259678 (PMC8580232; doi:10.1371/journal.pone.0259678)
Supplement: Attachment — Submitted filename: Response to Reviewers.docx [file pone.0259678.s002.docx]

**Rebuttal**

**Manuscript ID # PONE-D-21-16714**

**Title: Quantifying the Differential Consequences Between the Medial and Lateral Meniscus After Posterior Meniscus Root Tears**

We would like to thank the reviewers for their insightful comments and suggestions. We have taken the suggestions into consideration and revised the manuscript accordingly to address each of the reviewers’ questions and concerns.

**Editor Comments:**

1. Please ensure that your manuscript meets PLOS ONE's style requirements, including those for file naming. The PLOS ONE style templates can be found at
https://journals.plos.org/plosone/s/file?id=wjVg/PLOSOne_formatting_sample_main_body.pdf and
<https://journals.plos.org/plosone/s/file?id=ba62/PLOSOne_formatting_sample_title_authors_affiliations.pdf>.

**Author’s Response:**

We appreciate the editor’s critical feedback of the formatting requirements and we have reviewed the requirements provided in the above link. To address the concern, we have revised the manuscript accordingly.

**Author’s Action:**

We have changed the title to sentence case, added the location of the laboratory where the experiments were performed, removed the symbols denoting ‘current address’ as it is the same as the affiliation denoted by the numerical superscripts, and we have removed the corresponding author’s name and replaced it with ‘* Corresponding author,’ as described in the formatting requirement template.

2. Please include your full ethics statement in the ‘Methods’ section of your manuscript file. In your statement, please include the full name of the IRB or ethics committee who approved or waived your study, as well as whether or not you obtained informed written or verbal consent. If consent was waived for your study, please include this information in your statement as well.

**Author’s Response:**

We appreciate the editor’s critical feedback of the requirements for IRB or ethics approval. To address the concern, we have revised the manuscript by including the IRB approval and waiver of informed consent.

**Author’s Action:**

While the IRB approval organization and number was provided in the ethics statement in ‘editor manager,’ we have added the following to the beginning of the materials and methods section: “The University of Wisconsin-Madison Institutional Review Board approved the study under 2016-1316 and as a cadaveric study with anonymous data analysis written consent was not required.”

3. We note that the grant information you provided in the ‘Funding Information’ and ‘Financial Disclosure’ sections do not match.

When you resubmit, please ensure that you provide the correct grant numbers for the awards you received for your study in the ‘Funding Information’ section.

**Author’s Response:**

We appreciate the editor’s critical feedback of the requirements for the Funding Information and Financial Disclosure and we understand the importance of this information. To address this concern, we have revised the information accordingly and apologize for this confusion.

**Author’s Action:**

We have removed the funding information within the text of the manuscript that was previously under ‘Acknowledgements’ to comply with the formatting described within the pdf provided titled, “MANUSCRIPT BODY FORMATTING GUIDELINES.” The funding information provided within the online submission system is appropriate funding for this study. The additional funding information can be found in the revised cover letter.

4. Thank you for stating the following in the Acknowledgments Section of your manuscript:

Dr. Walczak was supported by NIH UW T32 AG000213-26, NIH UW TL1 TR000429, NIH UW UL1 TR000427, and UW-Madison Department of Orthopedic Surgery’s Freedom of Movement Award. No conflicts of interest, financial or otherwise, are declared by the authors.”

We note that you have provided funding information that is not currently declared in your Funding Statement. However, funding information should not appear in the Acknowledgments section or other areas of your manuscript. We will only publish funding information present in the Funding Statement section of the online submission form.

Please remove any funding-related text from the manuscript and let us know how you would like to update your Funding Statement. Currently, your Funding Statement reads as follows:

“B.E.W. was supported by NIH UW T32 AG000213-26, NIH UW TL1 TR000429, NIH UW UL1 TR000427, and UW-Madison Department of Orthopedic Surgery’s Freedom of Movement Award.”

Please include your amended statements within your cover letter; we will change the online submission form on your behalf.

**Author’s Response:**

Again, we appreciate and thank the editorial review process for this critical feedback and again we understand this importance of this information, both in regards to accuracy and format. To address this concern, we have revised the information accordingly and apologize for this confusion.

**Author’s Action:**

We have removed the funding information within the text of the manuscript that was previously under ‘Acknowledgements’ to comply with the formatting described within the pdf provided titled, “MANUSCRIPT BODY FORMATTING GUIDELINES.” We have placed in the ‘Acknowledgements’ section the person who assisted the work and she has approved her recognition in the acknowledgement section. Furthermore, the following is how we would like the Funding Statement to read, and is listed as ordered in the Funding Information section of the online submission site, “B.E.W. was supported by National Center for Advancing Translational Sciences under award numbers: UL1 TR000427, TL1 TR000429, and TL1 TR002375, National Institute on Aging under award number: T32 AG000213, and the Department of Orthopedic Surgery of the University of Wisconsin-Madison’s Freedom of Movement Award. The funders had no role in study design, data collection and analysis, decision to publish, or preparation of the manuscript.”

5. Thank you for stating the following in the Competing Interests section:

“I have read the journal's policy and the authors of this manuscript have the following competing interests: B.E.W. is a consultant for AlloSource and G.S.B. is a consultant for Conmed.”

Please confirm that this does not alter your adherence to all PLOS ONE policies on sharing data and materials, by including the following statement: ""This does not alter our adherence to  PLOS ONE policies on sharing data and materials.” (as detailed online in our guide for authors http://journals.plos.org/plosone/s/competing-interests).  If there are restrictions on sharing of data and/or materials, please state these. Please note that we cannot proceed with consideration of your article until this information has been declared.

Please include your updated Competing Interests statement in your cover letter; we will change the online submission form on your behalf.

**Author’s Response:**

Again, we appreciate and thank the editorial review process for this critical feedback regarding competing interests. We also want full transparency with any and all potential competing interests.

**Author’s Action:**

We have added the confirmation that any competing interests declared do not alter adherence to the PLOS ONE policies on sharing data and materials. We have also added into our cover letter the updated competing interest section as described and includes the following additional statement which accurately the author’s relationships, "This does not alter our adherence to  PLOS ONE policies on sharing data and materials.”

6. Thank you for stating the following in the Competing Interests section:

“I have read the journal's policy and the authors of this manuscript have the following competing interests: B.E.W. is a consultant for AlloSource and G.S.B. is a consultant for Conmed.”

We note that one or more of the authors are employed by a commercial company: Conmed

a. Please provide an amended Funding Statement declaring this commercial affiliation, as well as a statement regarding the Role of Funders in your study. If the funding organization did not play a role in the study design, data collection and analysis, decision to publish, or preparation of the manuscript and only provided financial support in the form of authors' salaries and/or research materials, please review your statements relating to the author contributions, and ensure you have specifically and accurately indicated the role(s) that these authors had in your study. You can update author roles in the Author Contributions section of the online submission form.

Please also include the following statement within your amended Funding Statement.

“The funder provided support in the form of salaries for authors B.E.W but did not have any additional role in the study design, data collection and analysis, decision to publish, or preparation of the manuscript. The specific roles of these authors are articulated in the ‘author contributions’ section.”

If your commercial affiliation did play a role in your study, please state and explain this role within your updated Funding Statement.

b. Please also provide an updated Competing Interests Statement declaring this commercial affiliation along with any other relevant declarations relating to employment, consultancy, patents, products in development, or marketed products, etc.

Within your Competing Interests Statement, please confirm that this commercial affiliation does not alter your adherence to all PLOS ONE policies on sharing data and materials by including the following statement: ""This does not alter our adherence to  PLOS ONE policies on sharing data and materials.” (as detailed online in our guide for authors http://journals.plos.org/plosone/s/competing-interests) . If this adherence statement is not accurate and  there are restrictions on sharing of data and/or materials, please state these. Please note that we cannot proceed with consideration of your article until this information has been declared.

Please include both an updated Funding Statement and Competing Interests Statement in your cover letter. We will change the online submission form on your behalf.

**Author’s Response:**

Again, we appreciate and thank the editorial review process for this critical feedback regarding competing interests. We also want full transparency with any and all potential competing interests.

**Author’s Action:**

We have added revisions to the cover letter as follows: “I have read the journal's policy and the authors of this manuscript have the following competing interests: B.E.W. is a consultant for AlloSource and G.S.B. is a consultant for Conmed. This does not alter our adherence to  PLOS ONE policies on sharing data and materials. The commercial affiliates had no role in study design, data collection and analysis, decision to publish, or preparation of the manuscript. The authors did not receive payment or renumeration in any form for any efforts related to this work.”

7. We note that you have stated that you will provide repository information for your data at acceptance. Should your manuscript be accepted for publication, we will hold it until you provide the relevant accession numbers or DOIs necessary to access your data. If you wish to make changes to your Data Availability statement, please describe these changes in your cover letter and we will update your Data Availability statement to reflect the information you provide.

**Author’s Response:**

We thank the editor’s response for repository information regarding the data. We have addressed the concerns and provided the relevant DOIs.

**Author’s Action:**

We have submitted the data to DRYAD and the data for this manuscript can be accessed by linking the following DOI: https://doi.org/10.5061/dryad.1ns1rn8v9.

8. Your ethics statement should only appear in the Methods section of your manuscript. If your ethics statement is written in any section besides the Methods, please delete it from any other section.

**Author’s Response:**

We understand and have revised the manuscript accordingly.

**Author’s Action:**

We added the ethical approval of this study in the first sentence in the methods section and removed from any other section.

Reviewers' comments:

Reviewer's Responses to Questions

Comments to the Author

1. Is the manuscript technically sound, and do the data support the conclusions?

The manuscript must describe a technically sound piece of scientific research with data that supports the conclusions. Experiments must have been conducted rigorously, with appropriate controls, replication, and sample sizes. The conclusions must be drawn appropriately based on the data presented.

Reviewer #1: Yes

**Author’s Response:**

We thank the reviewer for the positive comment.

Reviewer #2: Partly

**Author’s Response:**

We thank the reviewer for the essential feedback and address this concern below.

2. Has the statistical analysis been performed appropriately and rigorously?

Reviewer #1: Yes

**Author’s Response:**

We thank the reviewer for the positive comment.

Reviewer #2: I Don't Know

**Author’s Response:**

We thank the reviewer for their review.

**Author’s Action:**

To make complete assurances regarding the analyses, we have published the data from this study and it can be downloaded from the link provided above https://doi.org/10.5061/dryad.1ns1rn8v9.

3. Have the authors made all data underlying the findings in their manuscript fully available?

The PLOS Data policy requires authors to make all data underlying the findings described in their manuscript fully available without restriction, with rare exception (please refer to the Data Availability Statement in the manuscript PDF file). The data should be provided as part of the manuscript or its supporting information, or deposited to a public repository. For example, in addition to summary statistics, the data points behind means, medians and variance measures should be available. If there are restrictions on publicly sharing data—e.g. participant privacy or use of data from a third party—those must be specified.

Reviewer #1: Yes

**Author’s Response:**

We thank the reviewer for the positive comment.

Reviewer #2: No

**Author’s Response:**

We thank the reviewer for the feedback and we have now provided all data to DRYAD and is accessible on DOI: <https://doi.org/10.5061/dryad.1ns1rn8v9>.

4. Is the manuscript presented in an intelligible fashion and written in standard English?

PLOS ONE does not copyedit accepted manuscripts, so the language in submitted articles must be clear, correct, and unambiguous. Any typographical or grammatical errors should be corrected at revision, so please note any specific errors here.

Reviewer #1: Yes

**Author’s Response:**

We thank the reviewer for the positive comment.

Reviewer #2: Yes

**Author’s Response:**

We thank the reviewer for the positive comment.

5. Review Comments to the Author

Please use the space provided to explain your answers to the questions above. You may also include additional comments for the author, including concerns about dual publication, research ethics, or publication ethics. (Please upload your review as an attachment if it exceeds 20,000 characters)

Reviewer #1: Comment on the manuscript Number PONE-D-21-16714

Quantifying the Functional Consequences Between the Medial and Lateral Meniscus After Posterior Meniscus Root Tears

• The idea of the research is interesting for the readers of the journal. The authors used a cadaveric model to compare the effect of the meniscal root detachment of the medial and lateral meniscus on the meniscal extrusion. Using a cadaveric model, the test was carried out on both medial and lateral menisci during constant axial compression load, simulated axial load in various knee flexion angles (0-90°) as well as simulated non-weightbearing ROM (0-100°). The test was repeated while the meniscal root intact and after detaching the posterior meniscal roots.

**Author’s Response:**

We thank the reviewer for the positive comment.

• The study methodology is very clear. The results are reported in sufficient details. The statistical analysis used in the study is sound.

**Author’s Response:**

We thank the reviewer for the positive comment.

• The article is written in a clear and simple English language. I did not found any spelling or grammatical mistakes.

**Author’s Response:**

We thank the reviewer for the positive comment.

• With exception of figure 1, the included Diagrams and table are informative.

**Author’s Response:**

We thank the reviewer for the positive comment.

• The abstract is short and comprehensive and summarizes the important points of the article.

**Author’s Response:**

We thank the reviewer for the positive comment.

• The authors reported the weak points and the limitation of the study.

**Author’s Response:**

We thank the reviewer for the positive comment.

• Only few improvement suggestions:

­ line 195: I think the authors meant lateral meniscus not medial.

**Author’s Response:**

We thank the reviewer for their critical review of this. The typo is now corrected.

­ Figure 1 is of low quality and very low resolution/Pixel. For example you cannot read the flexion angle written in part C. I recommend to replace with pictures of higher resolution.

**Author’s Response:**

We thank the reviewer for their feedback and understand the concern and agree that the photographs can be improved.

**Author’s Action:**

Figure 1 has been revised with photographs of higher resolution for clarity. Additionally, we have added a high-resolution photo of the electronic goniometer capturing real-time angular measurements associated with meniscus extrusion. The figure legend has also been improved with more detail to illustrate the novel methods of this research.

• Decision:

According to the guidelines of the critical appraisal, I recommend to accept this Article for publication after considering the previously mentions improvement recommendations.

**Author’s Response:**

We thank the reviewer for their positive review.

Reviewer #2: Quantifying the Functional Consequences Between the Medial and Lateral Meniscus

After Posterior Meniscus Root Tears

Aim of the study:

The objective of this study was to understand essential differences in the response to posterior root tears (PRTs ) between the medial and lateral meniscus.

the authors conducted a cadaveric study to quantify the subluxation of both medial and lateral meniscus both with intact and with torn posterior root. This quantification was performed in 3 different settings; 1: axial compression load, 2: varying degrees of flexion (0-90°) with sinusoidal loading to mimic gait 3: dynamic flexion with no load (non weight bearing). The degree of subluxation was compared both between the intact and torn posterior horn within each meniscus and across both menisci.

This is a novel work with interesting findings. They provided a good introduction. The methodology was described in details. Statistical analysis was also described. Power calculation for sample size was conducted. The results are detailed and discussed adequately. Limitations of the study were also discussed.

points to be improved.

The title should be modified for example : Quantifying the differential Functional Consequences Between the Medial and Lateral Meniscus

After Posterior Meniscus Root Tears.

**Author’s Response:**

We welcome the reviewer’s critique and after considering the comments agree that the title should be modified to both more precisely characterize the purpose of this research and the findings.

**Author’s Action:**

To address this concern, we have revised the title to more precisely characterize the purpose of the research and subsequent findings. The title has been modified to, “Quantifying the differential functional behavior between the medial and lateral meniscus after posterior meniscus root tears.”

The abstract could be more simple and highlight the novelty of this work. It should be more attractive to let the reader continue into the article. The conclusion that functional consequences of extrusion are more significant

for the medial meniscus than that of the lateral meniscus however can not be simply made based on the degree of meniscus subluxation. Other factors that were not measured in this work such as changes in stress distribution across the joint surface with PRT could make such conclusion possible.

**Author’s Response:**

We thank the reviewer for their comments and agree that the abstract could better highlight the novelty of the work and be more attractive to reader to continue into the paper. We have revised the abstract in response to address this concern.

**Author’s Action:**

We have simplified the abstract to highlighting the novelty of the research and encouraging the reader to find details within the paper. Additionally, we have clarified the conclusion stating that there is a differential functional behavior in extrusion between the medial and lateral meniscus and this must be accounted for in posterior root repair.

The software used for statistical analysis should be mentioned

**Author’s Response:**

We agree that the software used for statistical analysis should be mentioned and we thank the reviewer for the suggestion.

**Author’s Action:**

We have added the software used for statistical analysis at the end of the ‘Statistical analysis’ subsection of the ‘Materials and methods’ section.

In the results section, how was the overall subluxation in table 1 measured? Please provide a supplementary table with all measures for all specimens.

**Author’s Response:**

We thank the reviewer for pointing out an area of needed clarification. We have revised the manuscript to provide full access to the data and the first paragraph in the ‘Results’ section was expanded to clarify how the overall subluxation was calculated.

**Author’s Action:**

To address this comment, we have provided the data using DRYAD accessible through https://datadryad.org/stash/share/XR2diRRfUYUd5cDw_d79mYO_i3iaJu0S5EW-FgcuMw8 and the manuscript was revised to include the doi number linked to the DRYAD repository where the full data is deposited and accessible upon publication of the research article in PLOS ONE at the end of the first paragraph in the ‘Results’ section. Table 1’s footnote was expanded to clarify the measurements of the Summary data.

Line 176, 177 : The greatest extrusion was recorded at 30-degrees of knee flexion (4.000 ± 01.259mm), please add the statistical significance . also in the same paragraph ensure that the statistical significance of each difference (which can be luckily found in the figures) is always mentioned

**Author’s Response:**

We agree with the reviewer that the narrative could be improved for clarity. We have revised the paragraph for the results regarding meniscus extrusion and knee flexion.

**Author’s Action:**

We have revised the figure 3 results narrative adding the statistical significance next to the point estimates and variation to augment the data provided in Figure 3.

Line 195: lateral meniscus instead of medial ?

**Author’s Response:**

We thank the reviewer for their critical review of this. The typo is now corrected.

In the conclusion, as mentioned above in the comment on the abstract, no functional consequences of extrusion were tested here, as for example the differential stress distribution across the joint in response to this measured meniscus subluxation. So the authors can not make this assumption. The direct fact that can be concluded here is obviously the significant subluxation of medial meniscus in the different given testing situations in comparison to the lateral meniscus.

**Author’s Response:**

We thank the reviewer for bringing up this point. We agree that in general the stress distribution across the joint is a useful piece of data. However, there are technical challenges in adding a sensor to measure stress distribution using this model, and we found that the sensor actually inhibited the dynamic motion of the meniscus in response to axial compression and range of motion, which was the focus of this work. Moreover, it is known that stress distribution and pressure is altered after detachment of the posterior root as many previous studies have demonstrated. For example, a seminal article showed that peak contact pressure after posterior root tear was similar to a total meniscectomy (Allaire et al., The Journal of Bone and Joint Surgery, 2008). A more recent study used a finite element analysis and demonstrated increased contact pressure and contact stress after posterior root avulsion (Wang et al., Journal of Orthopaedic Surgery and Research, 2021).

**Author’s Action:**

The discussion section was revised by adding a discussion point of what is known regarding the stress distribution and pressure changes associated with posterior root avulsions. We have also simplified the conclusion to highlight that focus of the work regarding the influence of knee motion and axial compression on the differential meniscus extrusion between the medial and lateral meniscus.

6. PLOS authors have the option to publish the peer review history of their article (what does this mean?). If published, this will include your full peer review and any attached files.

If you choose “no”, your identity will remain anonymous but your review may still be made public.

Do you want your identity to be public for this peer review? For information about this choice, including consent withdrawal, please see our Privacy Policy.

Reviewer #1: Yes: Ayman F. AbdelKawi

Reviewer #2: Yes: Dr. Mohammad Masoud

[NOTE: If reviewer comments were submitted as an attachment file, they will be attached to this email and accessible via the submission site. Please log into your account, locate the manuscript record, and check for the action link "View Attachments". If this link does not appear, there are no attachment files.]

While revising your submission, please upload your figure files to the Preflight Analysis and Conversion Engine (PACE) digital diagnostic tool, https://pacev2.apexcovantage.com/. PACE helps ensure that figures meet PLOS requirements. To use PACE, you must first register as a user. Registration is free. Then, login and navigate to the UPLOAD tab, where you will find detailed instructions on how to use the tool. If you encounter any issues or have any questions when using PACE, please email PLOS at figures@plos.org. Please note that Supporting Information files do not need this step.
